# Awareness and practices regarding eye diseases among patients with diabetes: a cross sectional analysis of the CoDiab-VD cohort

**DOI:** 10.1186/s12902-017-0206-2

**Published:** 2017-09-07

**Authors:** Lazaros Konstantinidis, Tania Carron, Eva de Ancos, Léonie Chinet, Isabelle Hagon-Traub, Emilie Zuercher, Isabelle Peytremann-Bridevaux

**Affiliations:** 1 Jules Gonin University Eye Hospital, University of Lausanne, Avenue de France 15 - Case Postale 5143 - 1000 Lausanne 2, Lausanne, Switzerland; 2 Institute of Social and Preventive Medicine (IUMSP), Lausanne University Hospital and University of Lausanne, Lausanne, Switzerland; 3 Private Practice, Morges, Switzerland; 4 Public Health Service, Department of Health and Social Action, Canton of Vaud, Lausanne, Switzerland

**Keywords:** Diabetes, Ocular complications, Diabetic retinopathy, Awareness, Practices

## Abstract

**Background:**

The increasing prevalence of diabetes is leading to a rise of eye diseases, augmenting the risk of sight-threatening complications. The aim of this study was to evaluate prevalence, awareness and practices regarding eye diseases among patients with diabetes in the canton of Vaud, Switzerland.

**Methods:**

A cohort of 323 patients with diabetes completed a self-administered questionnaire assessing prevalence, awareness and practices regarding eye diseases, besides health status and quality of care measures. Descriptive analyses followed by exploratory subgroup analyses and linear regressions were performed to investigate factors associated with awareness and practices.

**Results:**

While diabetic retinopathy was reported by 40.9% of patients with type 1 diabetes and 9.8% of patients with type 2 diabetes, 35.8% and 12.6% of all participants reported cataract and glaucoma, respectively. Awareness that diabetes could damage the eyes was reported by almost all participants; the majority was also aware of the importance of glycemic control and regular eye examination in preventing eye diseases. In contrast, only 70.5% of participants underwent an eye examination by an ophthalmologist during the past year. Eye examination was associated with better patients’ awareness. Barriers mentioned by patients revealed a lack of knowledge about screening guidelines, in particular regarding the preventive nature of eye examinations.

**Conclusions:**

Despite high levels of awareness regarding diabetic eye diseases, a significant proportion of patients with diabetes did not report annual eye examination. Both healthcare strategic efforts targeting the promotion of regular eye examination and initiatives aiming at improving knowledge of screening guidelines should be encouraged.

**Trial registration:**

ClinicalTrials.gov on 9th July 2013, identifier NCT01902043 (retrospectively registered).

## Background

Diabetes, a major public health problem affecting more than four hundred million people worldwide [1, 2], is associated with macrovascular and microvascular complications such as cerebrovascular diseases, kidney diseases, neural damages and eye diseases [3]. Among microvascular complications, vision loss from diabetic retinopathy [4],may be one of the most devastating complications for affected individuals. Currently, with the rising prevalence of diabetes [7], the latter complication emerges as a leading cause of avoidable visual impairment and blindness worldwide [8]. Additionally, other eye diseases that may compromise vision, such as cataract [5] and glaucoma [6], appear to increase in prevalence among patients with diabetes [7, 8].

Optimal management of diabetic retinopathy should include annual screening, adequate control of associated risk factors and timely treatment [4]. Furthermore, a significant element towards an optimal management, which is often undervalued, is the improvement of awareness and education among diabetic patients [9, 10]. Focusing on these parameters could enable actions targeting preventive strategies more effectively.

Previous studies assessing awareness and practices regarding eye diseases in patients with diabetes were mainly based in low- and middle-income countries, often reporting poor results [9–24]. Surprisingly, the association between poor awareness and/or practices and diabetic eye diseases has also been reported in high-income countries [8, 25–27].

Further and broader exploration and improved awareness and practices regarding eye diseases in patients with diabetes in general, not only focusing on diabetic retinopathy, may provide multiple benefits to patients and, consequently, to national healthcare systems. For example, it has been shown that ophthalmologic screening for patients with diabetes is highly cost-effective, compared to routinely provided medical interventions [28]. Furthermore, it was demonstrated that patients with diabetes could develop an expertise in the everyday management of their condition [29]. Finally, it has been reported that improving patient awareness of updated diabetes care recommendations and empowering them drives to better diabetes outcomes [30].

In that context, the aims of our study were first to describe eye diseases reported by patients with diabetes, second to assess their awareness and practices related to prevention of diabetic eye disease and, finally, to investigate potential determinants of patient awareness. To the best of our knowledge, this is the first study to evaluate awareness and practices regarding eye diseases among Swiss patients with diabetes, and one of very few studies conducted in a high-income country. In addition, whereas most other studies focused on diabetic retinopathy alone, we opted for a more comprehensive approach of eye diseases in patients with diabetes.

## Methods

### Study setting and study population

The CoDiab-VD cohort was launched in 2011–2012 in the canton of Vaud (a Swiss region of about 750,000 inhabitants). Non-institutionalized adults (≥18 years) reporting a diagnosis of diabetes for at least one year, residing in the canton of Vaud, with a sufficient level of French to understand and complete a self-report questionnaire, and without cognitive impairment or gestational diabetes were recruited through community-based pharmacies [31, 32]. Participants are followed-up annually since 2013 by self-administered paper-based postal questionnaire [32]. From the 519 patients with diabetes recruited in 2011–2012, 377 could be contacted in 2015, and 323 participants answered to the 2015 follow-up questionnaire and were included in our analyses.

### Study instrument

Patients’ self-reported outcomes harvested by postal questionnaire were considered. In 2015, the core questionnaire, described in detail elsewhere [32], was enriched with a thematic module entitled “Eyes and diabetes” that investigated eye diseases and treatments, eye examinations by an ophthalmologist along with their barriers and facilitators, as well as awareness of risk factors and prevention of diabetic eye diseases.

### Measures

The measures of interest for this study were i) patients’ characteristics and health status: age, gender, nationality (Swiss, other), education (primary, secondary, tertiary), civil status (married or living with a partner vs. other), economic hardship (difficulty in paying bills: yes, no; receipt of health insurance subsidies: yes, no), smoking status (current smoker vs. other), physical activity level using questions from the Swiss Health Survey (physically inactive vs. other) [33], ii) self-reported health status (excellent, very good, good, medium, poor), as well as iii) variables describing participants’ diabetes [type of diabetes (type 1, type 2, undetermined), duration of diabetes at the time of recruitment (≤10 years, >10 years), treatment (oral antidiabetic alone vs. insulin or other antidiabetic injection)], iv) participants’ membership in the local diabetes association (yes, no, do not know), v) the following outcome of care indicators: HbA1c awareness (yes, no, do not know), HbA1c value among HbA1c-aware patients, generic and diabetes-specific health-related quality of life measures (SF-12 physical and mental component scores – PCS and MCS, score range 0 = worst to 100 = best; Audit of Diabetes-Dependent Quality of Life 19 – ADDQoL, range − 9 = maximum negative impact of diabetes to +3 = maximum positive impact of diabetes), congruency of care with the Chronic Care Model (Patient Assessment of Chronic Illness Care – PACIC score, range 1 = lowest to 5 = highest congruency), and diabetes-related self-efficacy (Stanford Diabetes Self-Efficacy Scale, range 1 = lowest to 10 = highest self-efficacy), and also vi) process of care indicators (during the past 12 months) such as HbA1c check among HbA1c-aware patients (1×, ≥2×, none, do not know), blood pressure measurement (1×, 2-3×, ≥4×, none, do not know), lipid profile (yes, no, do not know), diabetic foot examination by a healthcare professional (yes, no, do not know), microalbuminuria test (yes, no, do not know), and influenza vaccination (yes, no, do not know); all latter processes of care variables were dichotomized (i.e. patients having had at least one check versus those not having had any, do not know answers not being considered).

The “Eyes and diabetes” 2015 thematic module targeted prevalence, awareness and practices regarding eye diseases, as well as barriers and facilitators to regular eye examination. First, patient-reported prevalence of eye diseases was measured with a multiple choice question including the following responses: diabetic retinopathy; cataract; age-related macular degeneration; myopia / hyperopia / astigmatism / presbyopia; other; no eye conditions; do not know; for those reporting diabetic retinopathy, treatment received was asked and the following response categories were available: laser therapy; eye injection; surgical intervention; other; no treatment; do not know. Second, we considered awareness, which refers to patients’ knowledge about diabetic eye diseases, and this was assessed with questions such as: prior knowledge that diabetes could damage the eyes (yes, no), knowledge of risk factors and prevention of diabetic eye diseases based on the following five items: maintaining good glycemic control; having regular eye examination by an ophthalmologist; maintaining good blood pressure control; maintaining good lipid control; nothing can be done, it is “bad luck”; (yes, no, do not know). The expected answers were “yes” for the four first items and “no” for the last one; this allowed the calculation of a score (referred to as the “awareness score” below) – for those answering at least 3 out of 5 items – constructed as the sum of the 5 items (each correct answer was given 1 point, otherwise 0) and ranging between 0 (= no correct answer) to 5 (= all answers correct). Then, we considered practices, which refers to the use of services regarding eye care, and was operationalized as the last eye examination (more precisely as the last dilated pupil examination of the eye fundus by an ophthalmologist) (0–12 months ago, 13–24 months ago, more than 24 months ago, never, do not know). We also asked patients, answering positively to a filter question on their awareness of the retinal photography examination, whether they had ever had a retinal photography examination (yes, without pupil dilatation; yes, with pupil dilatation; no; do not know). Finally, barriers and facilitators to regular eye examination by an ophthalmologist were also explored with a multiple choice question: i) barriers (no recommendation from the family physician or diabetologist; no information about diabetic eye diseases; no information about retinal screening; no time; financial reasons; too many other examinations and medical appointments; fear of the examination, result or treatment; fear of losing their driving license; discomfort during the examination (eye drops, dilated pupils); difficulty to find an ophthalmologist; difficulty to go to the ophthalmologist’s practice; no symptoms or vision problems; belief that it is not necessary because diabetes is well controlled; other) and ii) facilitators (recommendation of healthcare professionals; recommendation of relatives; feeling obliged to do it; knowledge of its importance; knowledge of the risks of a diabetes-related affection of the retina; knowledge of the treatment options; fear of having their eyes affected; having another eye problem necessitating an ophthalmologic follow-up).

### Statistical analysis

First, descriptive analyses of participants’ characteristics and eye-related variables were performed: means (SD) or percentages were computed for continuous or categorical variables respectively, as well as 95% confidence intervals for the prevalence of eye-related variables. Second, exploratory subgroup analyses were performed using T-test or chi-squared test, as appropriate. Finally, linear regressions were used to explore the association between the awareness score and explanatory variables a priori hypothesized to be associated with awareness: type of diabetes, age, gender, education, duration of diabetes at the time of recruitment, local diabetes association membership, presence of diabetic retinopathy, and eye examination by an ophthalmologist during the past two years. All analyses were performed using Stata 14.1.

### Ethical considerations

The study protocol was reviewed and approved by the Cantonal Ethics Committee of Research on Human Beings of the canton of Vaud (Protocol N° 151/11), and the CoDiab-VD cohort was registered with ClinicalTrials.gov, identifier NCT01902043. Written informed consent was obtained from all participants, and data are being kept confidential and anonymous.

## Results

### Characteristics of participants

Description of the study population is detailed in Table 1. In summary, mean age of respondents was 66.5 years and 38.7% were women. Whereas the majority (83.3%) reported type 2 diabetes, 57.5% were treated with insulin and/or other anti-diabetic injection. Also, while 18.4% were current smokers, 31% were physically inactive, and 18.3% declared being member of the local diabetes association.

**Table 1:** Participants characteristics

	Al ^a^	Type 1	Type 2
	(*n*= 323)	(*n* = 44)	(*n* = 269)
Mean (SD) age (years)	66.5 (10.6)	57.4 (14.0)	68.1 (9.3)
Women	38.7%	63.6%	34.9%
Nationality			
Swiss	83.5%	74.4%	85.9%
Other	16.5%	25.6%	14.1%
Education			
Primary	16.0%	14.3%	15.4%
Secondary	57.6%	54.8%	59.0%
Tertiary	26.4%	31.0%	25.6%
Married or living with partner	65.9%	56.8%	66.5%
Economic hardships			
Difficulties in paying bills during the past 12 months	22.8%	34.1%	20.3%
Health insurance subsidies	17.3%	20.5%	16.7%
Member of the local diabetes association^b^	18.3%	54.6%	12.7%
Current smoking	18.4%	14.0%	19.6%
Physically inactive	31.0%	29.6%	32.0%
Type of diabetes			
Type 1	13.6%	-	-
Type 2	83.3%	-	-
Undetermined	3.1%	-	-
Type of treatment			
Oral antidiabetics	42.6%	0.0%	50.8%
Insulin or other antidiabetic injection	57.5%	100%	49.3%
Self-reported health			
Excellent/very good	14.9%	21.4%	14.4%
Good	61.3%	64.3%	60.6%
Medium/poor	23.8%	14.3%	25.0%

^a^The 10 participants with an undetermined type of diabetes were not included in the subgroup analyses

^b^“Association Vaudoise du Diabète – AVD”

### Prevalence of eye conditions

As shown in Table 2, eye diseases were widespread among respondents. Besides common visual defects (i.e. myopia, hyperopia, astigmatism, presbyopia) that affected 36.2% of patients, cataract was the most frequently reported eye disease (35.8%). Whereas glaucoma was mentioned by 12.6% of patients, only 14.2% of patients reported being affected by diabetic retinopathy, a specific microvascular complication of diabetes. However, when looking at the disparity between types of diabetes, it appears that patients with type 1 diabetes reported being considerably more frequently affected by retinopathy than those with type 2 diabetes (40.9% vs. 9.8%). For those reporting diabetic retinopathy, this complication was reported to be mainly treated by laser therapy (75.6%), followed by intraocular injection (26.8%) and surgical intervention (19.5%). Up to 16% of patients with type 2 diabetes reported not being treated for their diabetic retinopathy, while this scenario was not reported by patients with type 1 diabetes. Finally, we observed that multiple eye diseases affected more often patients with type 1 diabetes than patients with type 2 diabetes (≥3 diseases: 20.9% vs. 4.7%).

**Table 2:** Prevalence of eye diseases as reported by patients

	All^a^		Type 1	Type 2
	(*n* = 323)		(*n* = 44)	(*n* = 269)
	%	[CI 95%]	%	%
Eye diseases ^b^ (*n* = 318)	(*n* = 318)		(*n* = 44)	(*n* = 265)
Diabetic retinopathy	14.2%	[10.3%–18.0%]	40.9%	9.8%
Cataract	35.8%	[30.5%–41.1%]	40.9%	35.1%
Glaucoma	12.6%	[8.9%–16.2%]	11.4%	12.8%
Age-related macular degeneration	4.7%	[2.4%–7.1%]	4.5%	4.5%
Myopia, hyperopia, astigmatism, presbyopia	36.2%	[30.9%–41.5%]	38.6%	35.5%
Other	2.8%	[1.0%–4.7%]	2.3%	3.0%
No	26.7%	[21.8%–31.6%]	22.7%	27.9%
Do not know	2.5%	[0.8%–4.2%]	2.3%	2.6%
Number of eye diseases reported (*n* = 310)	(*n* = 310)		(*n* = 43)	(*n* = 258)
0 disease	27.4%	-	23.3%	28.7%
1 disease	45.8%	-	37.2%	46.5%
2 diseases	20.0%	-	18.6%	20.2%
≥ 3 diseases	6.8%	-	20.9%	4.7%
Treatment for diabetic retinopathy (among patients reporting diabetic retinopathy) ^b^ (*n* = 41)	*n* = 41)		*n* = 15)	(*n* = 25)
Laser therapy	75.6%	[61.9%–89.3%]	100.0%	60.0%
Eye injection	26.8%	[12.7%–41.0%]	33.3%	24.0%
Surgical intervention	19.5%	[6.8%–32.2%]	26.7%	16.0%
Other	2.4%	[−2.5%–7.4%]	0.0%	4.0%
Retinopathy without having had treatment	9.8%	[0.3%–19.2%]	0.0%	16.0%
Do not know	2.4%	[−2.5%–7.4%]	0.0%	4.0%

^a^The 10 participants with an undetermined type of diabetes were not included in the subgroup analyses

^b^Multiple answers allowed

### Awareness regarding diabetic eye diseases

Regarding patients’ awareness about diabetic eye diseases and how to prevent them, a significant percentage of participants (96.0%) had prior knowledge that diabetes could damage the eyes; in fact, all patients with type 1 diabetes and 95.1% of patients with type 2 diabetes were aware of this risk. Furthermore, the vast majority of patients knew the benefit of maintaining good glycemic control and having regular eye examination by an ophthalmologist (98.6% and 97.5%, respectively) (Table 3). In contrast, benefits of maintaining good blood pressure control and good lipid control were less known, with respectively 91.3% and 85.4% of patients perceiving them as preventive behaviours. Finally a quarter of the participants answered positively to the item affirming that nothing can be done to prevent diabetic eye diseases, that’s the result of “bad luck”.

**Table 3:** Patients’ awareness about what can be done to prevent the occurrence or deterioration of diabetic eye diseases

	(*n*)	Expected answer	% of correct answers
Prevention means^a^			
Maintaining good glycemic control	(291)	Yes	98.6%
Having regular eye examination by an ophthalmologist	(284)	Yes	97.5%
Maintaining good blood pressure control	(288)	Yes	91.3%
Maintaining good lipid control	(274)	Yes	85.4%
Nothing can be done, it is “bad luck”	(236)	No	75.9%
Mean knowledge score [95%CI]^b^	(292)		4.1 [4.0–4.2]

^a^Results for patients having answered at least 3 out of the 5 items

^b^The score was constructed as the sum of the 5 items – for those answering to at least 3 out of 5 items; each correct answer was given 1 point, otherwise 0; range between 0 (= no correct answer) to 5 (= all answers correct)

### Practices: Frequency of eye examination

Seven out of ten patients (70.5%) reported having undergone an eye examination by an ophthalmologist during the past 12 months, and 87.0% during the past two years. Moreover, a very small proportion (3.7%) of respondents stated never having seen an ophthalmologist to screen for diabetic eye diseases. Furthermore, the proportion of patients visiting an ophthalmologist during the past two years was higher for patients with type 1 diabetes than for those with type 2 diabetes (95.5% vs. 85.5%). In addition, whereas retinal photography examination was known by 63.3% of patients, 83.9% of those answering positively to the previous question reported ever having undergone this examination, with or without pupil dilatation.

Patients who did not report an eye examination by an ophthalmologist during the past two years (13.0% of the total sample) differed from the rest of the sample on several points, while being similar regarding age, gender, education level and type of diabetes (Table 4). First, participants who did not report an eye examination during the past two years were less likely to report eye diseases (*p* = 0.001) or taking insulin or another antidiabetic injection (p = <0.001). Moreover, while they obtained a better disease-specific quality of life score (ADDQoL, *p* = 0.011), the care they reported was less congruent with the Chronic Care Model (PACIC: p = <0.001) than patients reporting an eye examination during the past two years. They also were less likely to be members of the local diabetes association (*p* = 0.009) or to be aware of what HbA1c is (p = <0.001). Finally, they reported having received globally less preventive care compared to the other respondents; some of these comparisons being statistically significant (HbA1c check: *p* = 0.047, foot examination: *p* = 0.003, influenza vaccination: *p* = 0.005).

**Table 4:** Subgroup analyses: comparison of patients visiting, or not, an ophthalmologist during the past 2 years

Visit to an ophthalmologist during the past 2 years		Yes (*n* = 280)	No (*n* = 42)	
	*n*	Mean (SD) or %	Mean or %	*p*-value^a^
Age	322	66.8 (10.5)	64.7 (11.5)	0.244
Gender	322			
Women		40.0%	31.0%	0.262
Men		60.0%	69.1%	
Education	317			
Primary		14.9%	23.8%	0.172
Secondary		59.6%	45.2%	
Tertiary		25.5%	31.0%	
Type of diabetes ^c^	322			
Type 1		15.0%	4.8%	0.153^c^
Type 2		81.8%	92.9%	
Undetermined		3.2%	2.4%	
Type of treatment	321			
Oral antidiabetic		37.9%	73.2%	<0.001
Insulin or other antidiabetic injection		62.1%	26.8%	
Member of the local diabetes association	321	20.1%	7.1%	0.053 ^c^
Number of eye diseases reported	310	1.2 (1.0)	0.6 (0.6)	0.001
HbA1C value^b^	174	7.3 (1.1)	6.8 (0.8)	0.313
ADDQoL global score	322	−1.5 (1.6)	−0.8 (1.1)	0.011
SF-12 PCS	312	43.5 (10.0)	44.8 (9.6)	0.440
SF-12 MCS	311	46.4 (11.1)	45.9 (10.2)	0.771
PACIC global score	316	2.8 (1.0)	2.2 (0.8)	<0.001
Stanford self-efficacy score	316	7.8 (1.6)	7.6 (1.9)	0.610
HbA1C awareness	303	91.3%	66.7%	<0.001
HbA1C check^b^	262	99.2%	92.0%	0.047 ^c^
Blood pressure measurement	315	99.3%	95.2%	0.087 ^c^
Lipid profile	311	97.4%	95.2%	0.349 ^c^
Diabetic foot examination	313	68.6%	45.2%	0.003
Microalbuminuria test	283	82.9%	71.1%	0.083
Influenza vaccination	314	67.7%	45.2%	0.005

^a^P-value from t-tests or Chi2/Fisher’s exact test

^b^Among HbA1C-aware patients

^c^Calculated with Fisher’s exact test

### Barriers and facilitators to regular eye examination

We then explored, with multiple choice questions, the reasons for patients to undergo or not a regular eye examination by an ophthalmologist (Table 5). The three main barriers to undergoing a regular eye examination mentioned by patients not screened during the past 12 months were the fact that they had no visual symptom or vision problem (32.9%), they found it unnecessary because their diabetes was well controlled (30.0%), and they did not get recommendations from their family physician or diabetologist (30.0%). Conversely, the three main facilitators for visiting regularly an ophthalmologist for eye screening, mentioned by patients regularly screened, were the fact that healthcare professionals recommended it (54.8%), that they were aware of the importance of regular controls (38.0%) as well as of the risks of diabetes-related affection of the retina (33.8%).

**Table 5:** Barriers and facilitators to regular eye examination by an ophthalmologist^a^

	%	[CI 95%]
Facilitators^b^	(*n* = 305)	
Recommendation of healthcare professionals	54.8%	[49.1%–60.4%]
Recommendation of relatives	2.3%	[0.6%–4.0%]
Feeling obliged to do it	9.8%	[6.5%–13.2%]
Knowledge of its importance	38.0%	[32.6%–43.5%]
Knowledge of the risks of a diabetes-related affection of the retina	33.8%	[28.4%–39.1%]
Knowledge of the treatment options	11.8%	[8.2%–15.4%]
Fear of having their eyes affected	22.3%	[17.6%–27.0%]
Having another eye problem necessitating an ophthalmologic follow-up	14.4%	[10.5%–18.4%]
Barriers^c^	(*n* = 70)	
No recommendation from the family physician or diabetologist	30.0%	[19.0%–41.0%]
No information about diabetic eye diseases	7.1%	[1.0%–13.3%]
No information about retinal screening	2.9%	[−1.1%–6.9%]
No time	10.0%	[2.8%–17.2%]
Financial reasons	4.3%	[−0.6%–9.1%]
Too many other examinations and medical appointments	4.3%	[−0.6%–9.1%]
Fear of the examination, result or treatment	1.4%	[−1.4%–4.3%]
Fear of losing their driving license	0.0%	-
Discomfort during the examination (eye drops, dilated pupils)	1.4%	[−1.4%–4.3%]
Difficulty to find an ophthalmologist	4.3%	[−0.6%–9.1%]
Difficulty to go to the ophthalmologist’s practice	4.3%	[−0.6%–9.1%]
No symptoms or vision problems	32.9%	[21.6%–44.1%]
Belief that it is not necessary because diabetes is well controlled	30.0%	[19.0%–41.0%]

^a^Multiple choice questions

^b^Results for patients having reported at least one eye examination (no time frame)

^c^Results for patients having not reported an eye examination during the past 12 months

### Exploratory analyses: Factors associated with patients’ awareness score

The exploratory linear regression analyses investigating the determinants of patients’ knowledge about prevention of diabetic eye diseases revealed that only one variable was associated with the awareness score: eye examination performed during the past two years (*p* = 0.005). No association was found with the seven others variables of the model (age, gender, education, type of diabetes, duration of diabetes at recruitment, member of the local diabetes association, and presence of diabetic retinopathy).

## Discussion

In this study, we explored patients’ reported prevalence, awareness and practices regarding eye diseases among patients with diabetes, as well as barriers and facilitators to regular eye examination. We found that eye diseases were frequent, especially among patients with type 1 diabetes. We also noticed that the majority of patients were aware of the positive influence of good glycemic control and of regular eye examinations by an ophthalmologist on the prevention of diabetic eye diseases, while the benefits of good blood pressure or good lipid controls were less well known. Also, whereas most participants underwent regular eye examinations by an ophthalmologist, one third of patients did not visit an ophthalmologist during the last year, mainly because they did not seem to be aware of the importance of preventive examinations. Finally, our exploratory model revealed that regular eye examination was positively associated with patients’ awareness of ocular preventive measures.

### Diabetes and eye diseases

Diabetes has been associated with the development of various eye diseases [34] including primarily retinopathy [4], but also cataract [5] and glaucoma. [6] More specifically, epidemiological studies have shown that the probability of retinal complications was higher in patients with type 1 than with type 2 diabetes: potentially vision-threatening retinal changes developed over time in up to 50% of patients with type 1 diabetes and 30% of those with type 2 diabetes [35]. In our study, we found that diabetic retinopathy, the leading cause of vision loss in adults aged 20–74 years [36], was reported by 40.9% of patients with type 1 diabetes and only 9.8% of patients with type 2 diabetes. Considering that diabetic retinopathy is generally present in about 30% of patients with type 2 diabetes [37], different hypotheses could explain this discrepancy. One could be that diabetic retinopathy is underestimated due to the fact that a percentage of patients had no eye examination during the last year. This inconsistency may also simply reflect poor knowledge of diabetic retinopathy status, an issue already shown in other studies [25, 27]. Additionally, it has been suggested that prevalence of retinopathy is likely underestimated in patients’ perceived history of diabetic retinopathy [38]. Moreover, considering that the vast majority of participants reporting diabetic retinopathy also reported having received ocular treatment for this condition, we may hypothesize that some participants falsely assume that they only present diabetic retinopathy if treatment is needed; for example in the presence of proliferative diabetic retinopathy or macular edema, therefore leading patients to under-report simple diabetic retinopathy. Another explanation might be that patients do not understand well what specialist physicians do and/or say during/after eye examination, and are consequently not well aware of their eye conditions. Correlations between patients and physicians reported annual eye and foot examinations suggest such an explanation [39]. The last explanation could be that our sample is not representative of the population of patients with diabetes.

We also found that cataract was reported by 35.8% of the participants, and by 40.9% of patients with type 1 diabetes despite the younger mean age of this group (57.4 years for patients with type 1 diabetes vs. 68.1 years for patients with type 2 diabetes). Epidemiologic studies have demonstrated that cataract is a common cause of visual impairment in patients with diabetes [40–42], and that patients with diabetes are 2–5 times more likely to develop cataract than their non-diabetic counterparts [42]. Since cataracts occur at an earlier age in patients with diabetes, it leads to a visual loss with a significant impact on the working population [5].

Finally, our results also showed that one in ten patients reported glaucoma; such results are in line with recent evidence suggesting that patients with diabetes are at greater risk of glaucoma [6], although it should be mentioned that the association between diabetes and glaucoma remains controversial for some authors [35]. This non-negligible percentage dictates the need for at least a basic glaucoma screening in patients with diabetes during their routine eye examination. In addition, as glaucoma evolves silently, with potentially irreversible and devastating visual loss when untreated, it may be wise to broaden the diabetes education perspective and include all significant diabetic eye diseases instead of focusing solely on diabetic retinopathy. .

### Awareness regarding diabetic eye diseases

In the present study, almost all patients with diabetes were aware that diabetes could damage the eyes, which is coherent with results from other studies conducted in high-income countries. For example, Schmid et al. reported that 96% of Australian patients with diabetes were aware that diabetes could be sight-threatening [43], and more than 98% of Japanese patients with type 2 diabetes were aware that diabetes could be related to eye damage [27].

Interestingly, our exploratory analyses revealed that eye examination by an ophthalmologist during the past 2 years was significantly associated with better awareness regarding diabetic eye diseases. This is not surprising considering that comprehensive eye examinations generally include patients’ education regarding ocular complications and guidance about preventive measures. These findings emphasize the need to promote regular examinations by an eye specialist.

### Practices: Frequency of eye examination

Despite a high level of awareness regarding diabetic eye diseases, about one third and one eighth of the participants did not report having had an eye examination performed by an ophthalmologist during the past 12 and 24 months, respectively. This divergence raises the question of the reasons for patients not to more frequently report undergoing eye examination. Our analysis of barriers and facilitators to regular eye examination provided some answers. Guidelines for the screening of diabetic retinopathy clearly state that, whether or not patients present ocular symptoms or unstable diabetes (i.e. poor glycemic control), a yearly eye examination is required since retinopathy may evolve silently [44]. However, one third of our participants reporting suboptimal screening practices believed that only the presence of ocular symptoms justifies an eye examination and one third that stable diabetes is enough to prevent diabetic eye diseases. Analogous results have been reported by others, with the main mentioned reason for skipping examination being the patients’ belief that they did not suffer from diabetic retinopathy [27]. It appears that patients tend to ignore the essentially preventive nature of regular eye examination, hence the necessity for healthcare professionals to emphasise this aspect with patients in the future.

The physicians’ potential role in improving practices also emerged from another barrier mentioned by one third of participants reporting insufficient eye examination: the absence of recommendations from their physician (family physician or diabetologist). The reinforcement of prevention messages delivered by physicians is required since appropriate guidance and information by primary care physicians alongside patient education and recommendations on eye health have been shown to be associated with higher adherence to screening guidelines [38, 45]. Better results could even be achieved knowing that physicians can positively impact screening rates by increasing patient referrals to eye specialists [39, 46].

Considering that the proportion of patients suffering from preventable eye diseases continues to rise and that screening for diabetic eye disease is one of the most cost-effective medical interventions in ophthalmology, initiatives aiming at improving adherence to screening recommendations are warranted [46]. To achieve this goal, both patients’ and healthcare professionals’ knowledge about screening guidelines need to be strengthened.

### Strengths and limitations

The strengths of our study lie in the fact that we explored the prevalence, awareness and practices regarding eye diseases in a population-based cohort of patients with diabetes whereas these concepts were previously investigated mainly in the context of ophthalmic consultations and limited to diabetic retinopathy. Also, it considered several key measures and outcomes of interest in the field of diabetes care. Nevertheless our results need to be interpreted in consideration of the following limitations. First, they were based on patients’ self-reported data, which may be prone to recall as well as desirability bias. However, previous analyses of the Codiab-VD cohort data showed good agreement between patients- and physician-reported outcomes [47]. In addition, self-reported patient-centered outcomes are increasingly considered as key in the evaluation of patients’ care and therefore need to be used. Secondly, despite the fact that the Co-Diab-VD participants may not be representative of the general population of patients with diabetes, patients recruited in the CoDiab-VD cohort appeared to share similar characteristics with participants in other studies conducted in the same region [48, 49].

## Conclusion

In conclusion, the level of awareness regarding diabetic eye diseases appeared to be relatively high in patients with diabetes residing in the canton of Vaud, Switzerland, while there is still room for improvement in diabetic eye diseases screening practices. Barriers mentioned by patients revealed their lack of knowledge about screening guidelines, in particular regarding the preventive nature of eye examinations. Consequently, diabetes-related ocular screening guidelines for patients and healthcare professionals should be promoted in order to reduce patients’ misconceptions and help them change practices for earlier detection of diseases and, finally, reduce the risk of sight-threatening complications.
